# IL-33 and its increased serum levels as an alarmin for imminent pulmonary complications in polytraumatized patients

**DOI:** 10.1186/s13017-019-0256-z

**Published:** 2019-07-19

**Authors:** Gabriel Halát, Thomas Haider, Michel Dedeyan, Thomas Heinz, Stefan Hajdu, Lukas L. Negrin

**Affiliations:** University Department of Orthopedics and Trauma-Surgery, Medical University of Vienna, Währinger Gürtel 18-20, 1090 Vienna, Austria

**Keywords:** IL-33, Alarmin, Polytrauma, ARDS, Biomarker, Thoracic injury

## Abstract

**Background:**

According to recently published findings, we hypothesized that serum interleukin-33 (IL-33) may qualify for predicting pulmonary complications in polytraumatized patients.

**Methods:**

One hundred and thirty patients (age ≥ 18 years, ISS ≥ 16) were included in our prospective analysis after primary admission to our level I trauma center during the first post-traumatic hour. Serum samples immediately after admission and on day 2 after trauma were obtained and analyzed.

**Results:**

Median initial IL-33 levels (in picograms per milliliter) were higher in polytrauma victims (1) with concomitant thoracic trauma [5.08 vs. 3.52; *p* = 0.036], (2) sustaining parenchymal lung injury (PLI) [5.37 vs. 3.71; *p* = 0.027], and (3) developing acute respiratory distress syndrome (ARDS) [6.19 vs. 4.48; *p* = 0.003], compared to the respective rest of the study group. The median initial IL-33 levels were higher in patients experiencing both PLI and ARDS compared to those sustaining PLI and not developing ARDS [6.99 vs. 4.69; *p* = 0.029]. ROC statistics provided an AUC of 0.666 (*p* = 0.003) and a cut-off value of 4.77 (sensitivity, 71.8%; specificity, 75.7%) for predicting ARDS. Moreover, a higher initial median IL-33 level was revealed in the deceased compared to the survivors [12.25 vs. 4.72; *p* = 0.021]. ROC statistics identified the initial level of IL-33 as a predictor of death with 11.19 as cut-off value (sensitivity, 80.0%; specificity, 80.0%; AUC = 0.805; *p* = 0.021).

**Conclusions:**

Following tissue damage, IL-33 is abundantly released in the serum of polytraumatized patients immediately after their injuries occurred. As initial IL-33 levels were particularly high in individuals experiencing both PLI and ARDS, IL-33 release after trauma seems to be involved in the promotion of ARDS and might serve already at admission as a solid indicator of impending death in polytraumatized patients.

## Background

In polytrauma care, the timing of definitive surgical treatment of major skeletal injuries is crucial for morbidity and mortality. In general, there are two treatment strategies for fracture care in polytraumatized patients [1]. Early total care involves definitive surgical stabilization of all long bone fractures during the early phase of treatment, whereas damage control orthopedics suggests temporary external fracture fixation with secondary definitive osteosynthesis after stabilization of the patient’s physiological and immunological status at the intensive care unit (ICU). Polytrauma victims with concomitant severe chest trauma are at high risk of developing an acute respiratory distress syndrome (ARDS) and/or pneumonia. As these patients are considered especially vulnerable to a sustained inflammatory response from immediate surgical intervention, damage control orthopedics seems to be their treatment of choice as it reduces the risk for complications and adverse outcome related to the surgical second hit [2]. However, pulmonary complications may develop while waiting for secondary surgery, potentially compromising definitive osteosynthesis, and thus the best possible outcome. In consequence, the choice of fracture treatment poses a serious challenge to trauma surgeons. Providing decision-making tools concerning this matter was the impetus of our biomarker research.

**Fig. 1:**
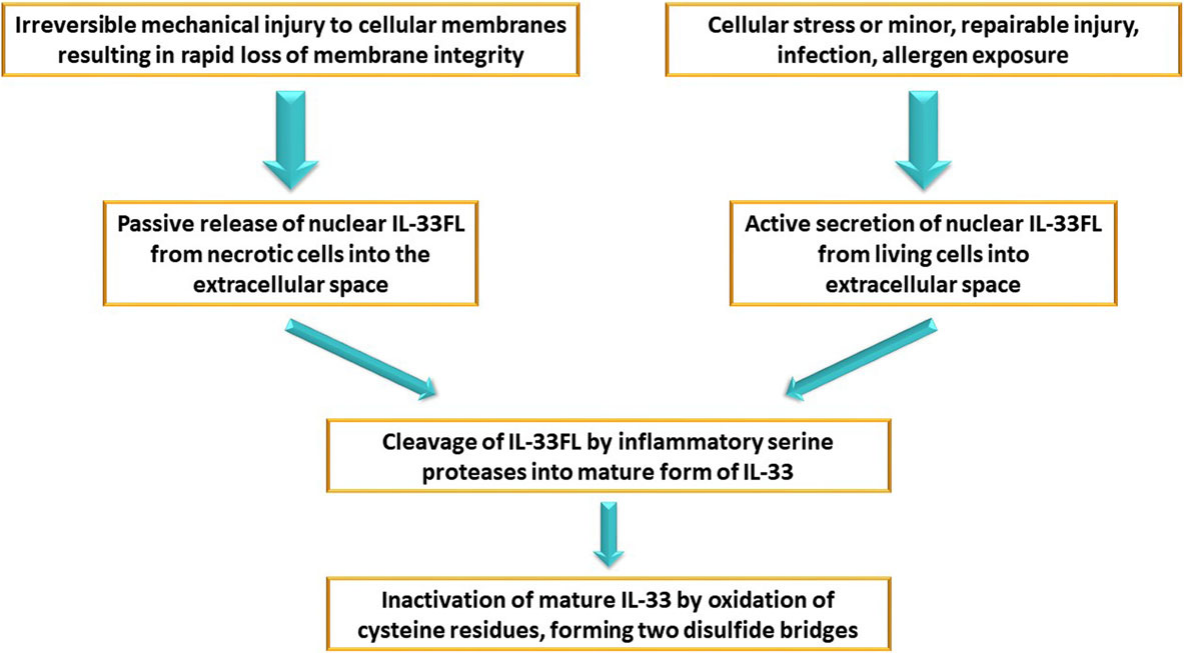
Release/secretion and inactivation mechanism of IL-33

Interleukin-33 (IL-33) is the most recent addition to the IL-1 family, discovered in 2005 [3]. Full-length IL-33 (IL-33FL) is composed of a nuclear domain, which is critical for nuclear localization and chromatin association, a connecting central part, and an IL-1-like cytokine domain, which is essential for activation of the immune system [4, 5]. It is constitutively expressed at high basal levels and stored within the nuclear compartment of structural cell types, most notably by vascular endothelial cells and epithelial cells of human barrier tissues [3, 4]. According to the current knowledge, passive release and active secretion make nuclear IL-33FL available to the extracellular space [4, 5], where it is processed into shorter mature forms [6]. They contain an intact IL-1-like cytokine domain and exhibit an up to 30 times greater biological activity than IL-33FL, depending on the size of the protein [6, 7] and are inactivated within 2 h in the extracellular environment [4, 8]. The relevant mechanisms are presented in Fig. 1 [5, 6, 9–13].

IL-33 has been identified as an endogenous alarm signal (alarmin) [11, 14] to alert various types of immune cells to tissue damage or stress [3, 15, 16]. Both full-length and mature IL-33 (hereafter collectively referred to as IL-33) act specifically through the receptor ST2L, a transmembrane, long isoform of suppression of tumorigenicity 2 (ST2) [17, 18], whose expression is restricted to the surface of Th2 cells and mast cells [18]. Whereas ST2L mediates the inflammatory effect of IL-33, sST2, a short, soluble isoform, which is mainly secreted by fibroblasts [19], exerts immunosuppressive activity by acting as a decoy receptor that prevents the interaction of ST2L with IL-33 [20] or shows a direct anti-inflammatory impact [21].

To the present day, only a few studies have been published on the role of IL-33 in ARDS. Using a mouse model, Fu and coworkers identified IL-33 as an important factor involved in the progression of pulmonary inflammation in ARDS [22]. Lin and coworkers detected higher serum levels of IL-33 in 14 patients suffering from ARDS compared to healthy controls [23].

The objective of our study was to analyze serum IL-33 levels in severely injured patients and investigate its potential role as an alarmin in polytrauma care. Considering previously published data on IL-33, we were further interested whether IL-33 is involved in pulmonary complications commonly observed in this patient collective.

## Methods

From 2011 to 2015, after approval by the Local Ethics Committee of the Medical University of Vienna (project number 368/2011), we included 130 polytrauma victims [Injury Severity Score (ISS) ≥ 16; age ≥ 18 years] into our study population. All of them were directly admitted to our level I trauma center within 1 h after trauma and were transferred to the intensive care unit after initial treatment due to a compromised medical condition surviving for at least 24 h. Burn victims and patients with known malignancies or chronic inflammatory lung diseases were excluded.

One additional separating gel tube (Vacuette® 4 mL; Greiner Bio-One International) for biomarker level assessment was collected during routine blood work at admission and on day 2 (24–48 h) after trauma. These samples were immediately centrifuged at 3000×*g* for 15 min at room temperature. Sera were removed and stored at − 80 °C until further assessment. Analysis was performed only in patients, where informed consent could be obtained from the patient. In deceased patients, informed consent was obtained either from the patient prior to death or the closest relative, or from the patient’s legal representative.

Initially, the collected samples were used for the measurement of five biomarker levels [24]. Remaining serum aliquots were analyzed for this post hoc study. Serum levels of IL-33 were measured using an enzyme-linked immunosorbent assay (Human IL-33 ELISA Kit, Promokine PK-EL-62958 Minneapolis, MN, USA) according to the manufacturer’s instructions. All samples were evaluated in triplets and the mean values were calculated. To obtain a reference value, only one blood sample was taken from 10 age-matched, healthy individuals.

Parenchymal lung injury (PLI) was detected by computed tomography scans performed at admission. ARDS was diagnosed according to the Berlin definition [25], which is based on clinical and radiographic data. Pneumonia was identified by a temperature deviation from normal (> 38 °C or < 35.5 °C), either leucocytosis (white cell count > 10,000/mm^3^ or > 10% immature forms) or leucopenia (white cell count < 4,000/mm^3^); a macroscopically purulent sputum, the presence of a newly developed cough, dyspnea, and/or tachypnea (in the case of spontaneous breathing patients); and a new or changing infiltrate on chest radiographs.

Statistical analysis was conducted using IBM SPSS Statistics (Version 24). Due to skew distributions, parameters are presented as median and interquartile range in round brackets. Continuous variables were matched using the Mann-Whitney-Wilcoxon rank-sum test (for unrelated samples) and the Wilcoxon signed-rank test (for related samples). Categorical data were analyzed by means of the chi-square test. Receiver operating characteristic (ROC) curves and areas under the curve (AUC) were computed, the latter presented with a 95% confidence interval (CI). Cut-off values were determined by the maximum sum of sensitivity and specificity [26]. For correlations, the Spearman rank coefficient (*ρ*) was calculated. In general, the threshold of significance was set at *p* = 0.05.

## Results

Demographic data of our polytraumatized patients are presented in Table 1. ARDS onset was observed within 2 days after admission in 32 of the affected 42 patients, whereas first signs of pneumonia were clearly distinct on day 4 after admission. This was even the case in patients with primary onset of ARDS. In all individuals suffering from pneumonia and ARDS, pneumonia was diagnosed after the occurrence of ARDS. During hospitalization, five patients died after a median time period of 3 days (range, 1–24); causes of death were ARDS in three cases and one case of multiorgan failure and traumatic brain injury, respectively.

**Table 1:** Demographic data

	Median/number
Age	39 (26–55) years
Male/female	90/40
ISS	30 (22–41)
Thoracic trauma	118
PLI	108
ARDS	42
Pneumonia	41
ARDS and pneumonia	27
ARDS no pneumonia	15
Pneumonia no ARDS	14
No ARDS no pneumonia	74
C-reactive protein day 0	0.16 (0.05–0.47) g/L
C-reactive protein day 2	6.26 (2.38–10.19) g/L
Leukocyte count day 0	12.22 (9.39–16.32) g/L
Leukocyte count day 2	9.05 (7.07–10.76) g/L
Hemoglobin concentration day 0	11.70 (9.48–13.30) g/L
Hemoglobin concentration day 2	9.90 (8.80–11.40) g/L

IL-33 levels assessed at admission and on day 2 after trauma are presented in Table 2.

**Table 2:** IL-33 levels

		IL-33 initial (pg/mL)	IL-33 day 2 (pg/mL)	*p* value
Total		4.81 (3.39–9.20)	3.45 (2.07–5.02)	< 0.0001
Gender	Male	4.99 (2.97–8.83)	3.51 (2.00–5.04)	< 0.0001
	Female	4.72 (3.71–9.72)	3.30 (2.12–4.93)	< 0.0001
		*p* = 0.779	*p* = 0.911	
Thoracic trauma	Yes	5.08 (3.48–9.61)	3.51 (2.02–5.05)	< 0.0001
	No	3.52 (2.51–4.70)	2.59 (2.17–4.34)	0.012
		*p* = 0.036	*p* = 0.545	
PLI	Yes	5.37 (3.48–9.71)	3.58 (2.11–5.03)	< 0.0001
	No	3.71 (2.43–5.17)	2.33 (1.89–3.87)	0.001
		*p* = 0.027	*p* = 0.083	
ARDS	Yes	6.19 (4.42–11.90)	4.70 (2.79–6.36)	< 0.0001
	No	4.48 (2.79–6.33)	2.79 (1.64–4.40)	< 0.0001
		*p* = 0.003	*p* = 0.001	
Pneumonia	Yes	5.41 (3.83–10.81)	3.94 (2.49–5.60)	< 0.0001
	No	4.58 (3.07–7.90)	3.04 (1.72–4.74)	< 0.0001
		*p* = 0.106	*p* = 0.219	
Fatality	Yes	12.25 (7.58–23.68)	3.53 (2.96–15.48)	0.068
	No	4.72 (3.27–8.25)	3.42 (2.92–5.01)	< 0.0001
		*p* = 0.021	*p* = 0.430	

In all of our patients, initial IL-33 levels were higher than the reference value of 0.25 pg/mL provided by our healthy controls. In our study group and all respective subgroups median IL-33 levels declined from admission to day 2 after trauma. Of interest, only in 15 individuals (11.5%) an increase in IL-33 levels could be observed. Comparing patients with an increase and decrease of IL-33 levels within the first two post-traumatic days revealed solely a higher leukocyte count in the former group [15.99 (10.48–19.34) g/L vs. 11.86 (8.92–15.40) g/L]. Furthermore, a strong correlation (*ρ* = 0.721; *p* < 0.0001) between the initial and the day 2 levels of IL-33 was observed.

In order to identify the individuals presenting with the highest IL-33 levels at admission, we subdivided our polytraumatized patient collective according to the parameters PLI and ARDS, resulting in four groups: “−PLI−ARDS” combing patients without PLI and ARDS, “−PLI+ARDS” denoting those without PLI and suffering from ARDS as well as “+PLI−ARDS,” and “+PLI+ARDS” including those sustaining PLI and not developing/developing ARDS. Significant differences in AIS_Thorax_, ISS and the development of pneumonia are presented in Table 3.

**Table 3:** Subgroup characteristics

		Groups				
		−PLI−ARDS	−PLI+ARDS	+PLI−ARDS	+PLI+ARDS	*p* value
AIS_Thorax_	0	9	3	0	0	< 0.0001
	1	3	0	0	0	
	2	4	0	5	5	
	3	3	0	33	4	
	4	0	0	21	14	
	5	0	0	10	16	
Pneumonia	Yes	4	3	10	24	< 0.0001
	No	15	0	59	15	
ISS		22 (17–34)*	29 (26–34)	29 (22–38)	36 (26–43)*	0.003*

*Tag the two groups with significantly different ISS values

As the three individuals forming the “−PLI+ARDS” did not show any pathologic changes in their thoracic CT scans, they suffered from indirect ARDS. Of interest, no statistically significant differences in initial IL-33 levels between the “−PLI+ARDS” and the “+PLI+ARDS” group could be calculated [2.64 (2.35–2.64) pg/mL vs. 6.99 (4.56–12.17) pg/mL; *p* = 0.172].

**Fig. 2:**
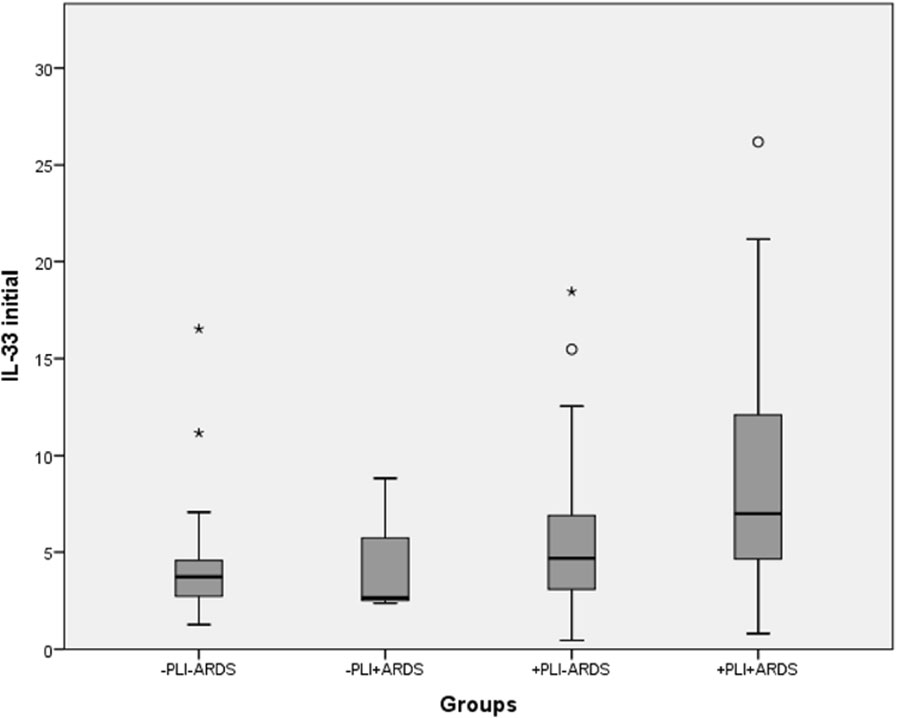
Distribution of initial IL-33 levels according to group assignment

Figure 2 graphically displays the distribution of initial IL-33 levels in the four subgroups. Significant differences could be revealed solely between the “−PLI−ARDS” and the “+PLI+ARDS” group (*p* = 0.009) as well as between the “+PLI−ARDS” and the “+PLI+ARDS” group (*p* = 0.029).

**Fig. 3:**
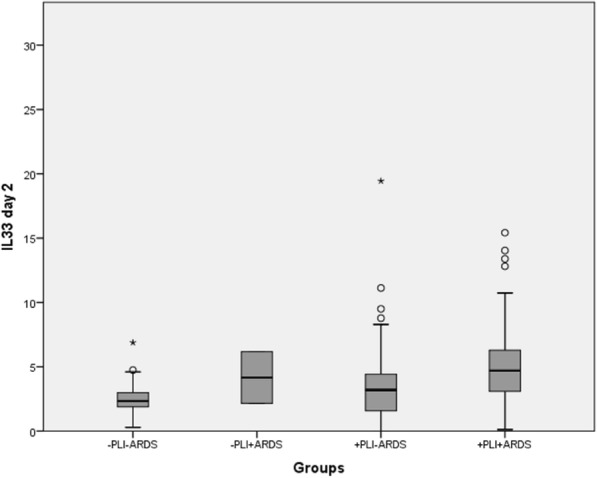
Distribution of IL-33 day 2 levels according to group assignment

The distribution of IL-33 levels assessed in the four subgroups on day 2 after trauma is presented in Fig. 3. Significant differences between the “−PLI−ARDS” and the “+PLI+ARDS” group (*p* = 0.023) as well as between the “+PLI−ARDS” and the “+PLI+ARDS” (*p* = 0.039) were detected.

**Fig. 4:**
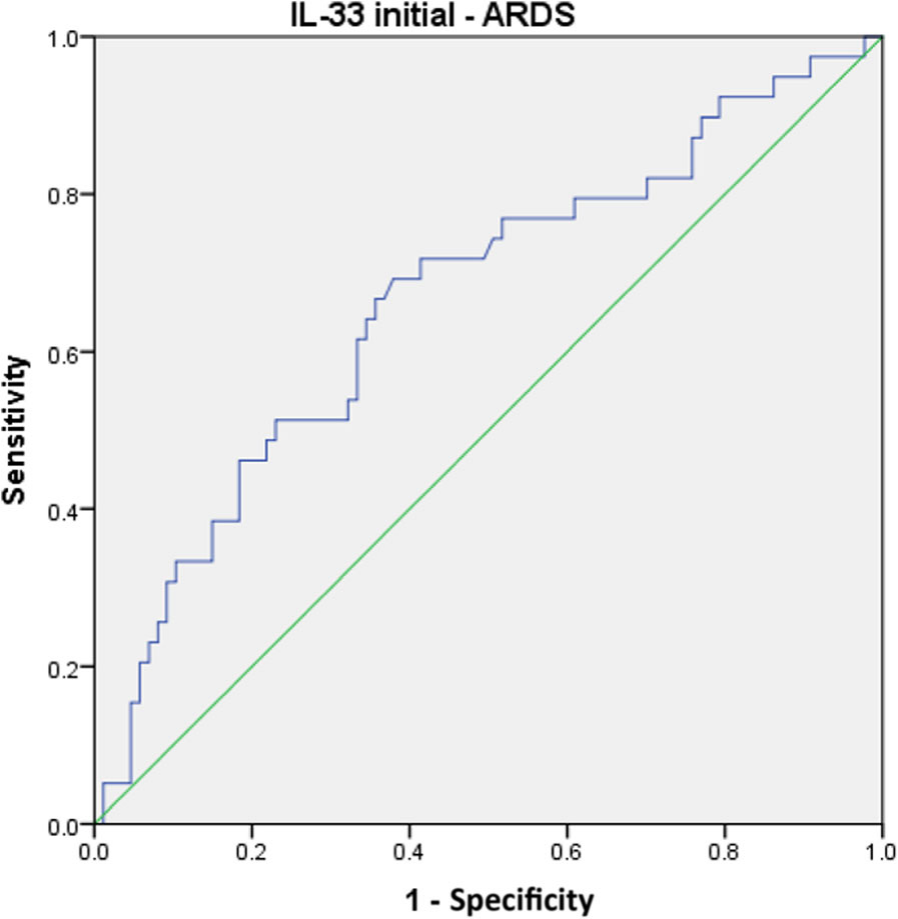
ROC curve for the initial IL-33 level and ARDS

Due to the fact that ARDS was diagnosed in 76.2% of the affected patients up to day 2, solely initial IL-33 levels may serve as a predictor of ARDS in clinical practice. The corresponding ROC curve is shown in Fig. 4, providing an AUC of 0.666 (95% CI, 0.561–0.771; *p* = 0.003) and a cut-off value of 4.77 pg/mL (sensitivity, 71.8%; specificity, 75.7%).

**Fig. 5:**
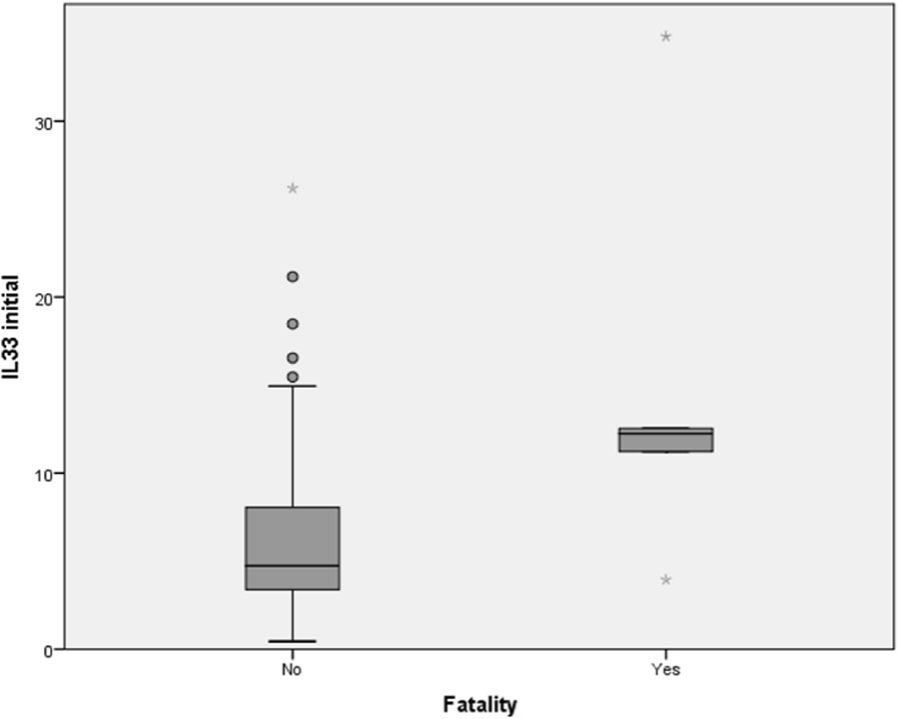
Distribution of initial IL-33 in survivors and deceased

As Table 2 shows, initial IL-33 levels were 2.6 times higher in deceased patients compared to survivors with their distributions being displayed in Fig. 5. However, no significant difference could be revealed on day 2 in this regard.

**Fig. 6:**
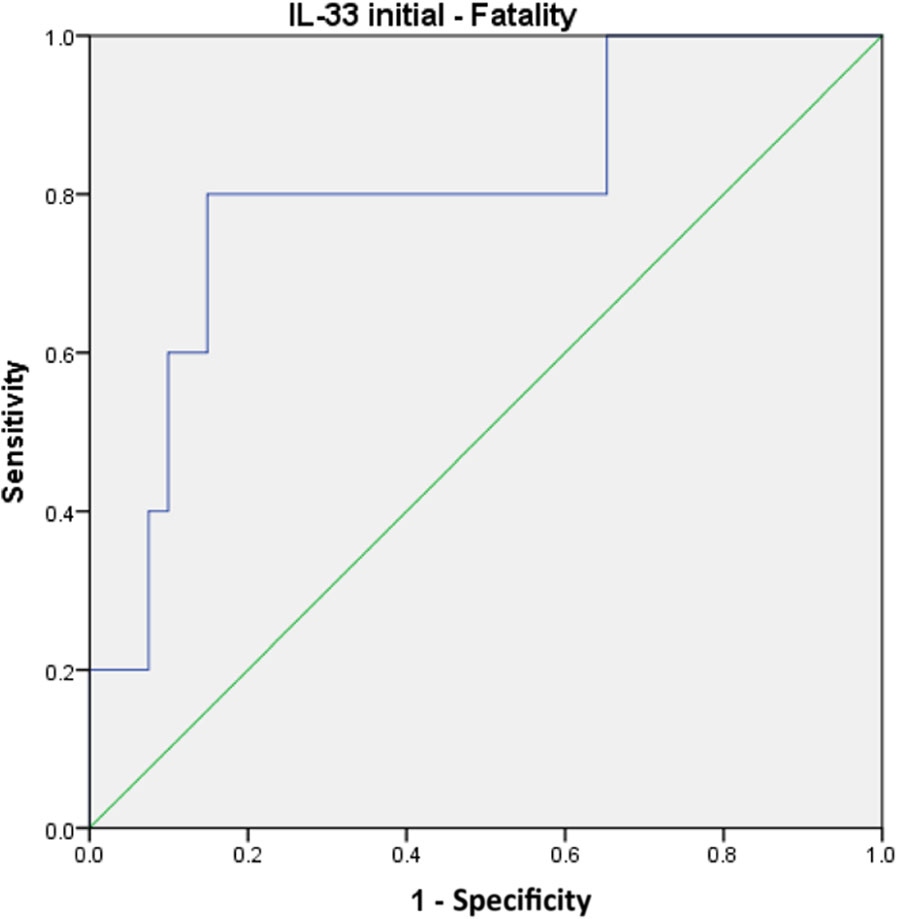
ROC curve for the initial IL-33 level and fatality

ROC statistics identified the initial IL-33 level as a predictor of death with an AUC of 0.805 (95% CI, 0.596–1.000; *p* = 0.021) and a cut-off value of 11.19 pg/mL (sensitivity, 80.0%; specificity, 80.0%). The respective ROC curve is presented in Fig. 6.

Finally, we focused on correlations between the initial and the day 2 levels of IL-33 and all continuous parameters assessed in this study, including initial lactate and C-reactive protein levels. Initial IL-33 levels were solely significantly correlated to ISS (*ρ* = 0.274; *p* < 0.01), duration of mechanical ventilation (*ρ* = 0.298; *p* < 0.01), and hospitalization period (*ρ* = 0.212; *p* < 0.05), whereas day 2 levels of IL-33 were only significantly correlated to the duration of mechanical ventilation (*ρ* = 0.365; *p* < 0.01).

## Discussion

To our knowledge, we are the first to evaluate IL-33 levels as a potential biomarker in polytraumatized patients with respect to its predictive value on secondary pathophysiological conditions. Our prospective study revealed a generally sharp increase in IL-33 levels after polytrauma. Both initial and day 2 levels of IL-33 were significantly higher in polytraumatized patients sustaining PLI and developing ARDS compared to those only sustaining PLI, and, clinically most relevant, initial IL-33 levels higher than 11.19 pg/mL were identified to indicate a high risk of death following polytrauma.

PLI is characterized by micro-hemorrhages caused by alveolar damage and alveolar strain that occur at traumatic separation of alveoli from airway structures and blood vessels created by shear stress or overdistension [27]. As the internal surface of the alveoli is lined by the epithelium [28], local regions of high mechanical stress are likely to cause direct necrosis of alveolar epithelial cells [29]. The interaction of PLI-induced tissue injury and inflammatory response results in an increase of cell membrane permeability, protein-rich alveolar edema, and increased epithelial cell necrosis [30, 31], all of which potentially promote the development of direct ARDS [32]. Whereas the latter is caused by direct injury to the lung epithelium, in indirect ARDS, the vascular endothelium of the lung is diffusely damaged by circulating inflammatory mediators released in the setting of systemic disorders [33].

Meeting the expectations of an alarmin in the event of a barrier breach, IL-33 was abundantly detected in the serum of polytrauma victims already at admission. The lung was identified as not the only, but a potential relevant source of IL-33 as its levels were significantly higher in individuals with concomitant thoracic trauma. Due to the fact that 91.5% of them sustained PLI, our study group was subdivided according to its presence. Not surprisingly, initial IL-33 levels were higher in the PLI group. As initial IL-33 levels were also higher in the ARDS group compared to the non-ARDS group, we subdivided our patients according to the parameters “presence of PLI” and “development of ARDS,” revealing a significant difference in initial IL-33 levels between the “+PLI−ARDS” group and the “+PLI+ARDS” group. Justified by its abundant storage in the nuclei of parenchymal cells, the major proportion of IL-33 in our opinion was most likely released during necrosis. Because extensive epithelial necrosis is a prominent feature of direct ARDS in the current state of knowledge [32], the high levels of initial IL-33 in the “+PLI+ARDS” group might mainly originate from irreparably, mechanically damaged epithelial cells of the lung. Moreover, we hypothesize that IL-33 release may be an initial event in the inflammatory process of direct ARDS. As the pathogenesis of indirect ARDS starts with endothelial damage [33], differences in initial IL-33 levels between patients developing direct and indirect ARDS have to be expected. Whereas Lin and coworkers revealed significantly higher IL-33 levels in patients suffering from direct ARDS compared to those with indirect ARDS (*p* < 0.01) [23], our marked difference (6.99 pg/mL vs. 2.84 pg/mL) did not reach statistical significance, probably due to the group size mismatch.

According to ROC statistics IL-33 levels, which are assessed at admission and exceed the cut-off value of 4.77 pg/mL, they accurately identify 71.8% of polytraumatized patients developing ARDS, whereas 75.7% of the individuals with an initial IL-33 level lower than 4.77 pg/mL are expected not to suffer from ARDS. Unfortunately, our cut-off value is not statically robust enough to identify initial IL-33 as a reliable predictor of ARDS; nevertheless, it might yield distinct information for the clinician. IL-33 release is not lung-specific; it is also expressed at epithelial surfaces in the skin, stomach, intestine, salivary gland, vagina, and lung [34]. Moreover, transient release of endogenous IL-33 has been reported after mechanical skin injury [35] and, because of its widespread expression in the nuclei of endothelial cells from blood vessels along the vascular tree, IL-33 can be found in almost all human organs [3]. Due to the multitude of possible sources of IL-33 in the human body, the high initial IL-33 levels may indicate alarming rates of necrotic cell death in several vital organs, thus alerting a compromised patient condition at admission that necessitates damage control orthopedics. Early total care, associated with a prolonged surgery time, may induce an even more compromising secondary impact on the injured organism. However, using our cut-off value of 11.19 pg/mL, we were able to identify a lethal outcome in polytraumatized patients with an accuracy of 80%, supporting our hypothesis.

As expected of an alarmin, IL-33 levels declined in 96.2% of our patients within the first two post-traumatic days. The rapid inactivation of the released protein, which limits its range and duration of action, in combination with an increasingly smaller release from necrotic cells might be the reason for this finding.

## Conclusions

As serum IL-33 levels increased in all of our patients immediately after they had sustained multiple injuries, IL-33 can be considered as an alarmin in the response to polytrauma, indicating the amount of damaged structural cells by mechanical impact. Particularly high initial IL-33 levels were detected in individuals experiencing both PLI and ARDS, probably due to the highest amount of necrotic epithelial lung cells. Although we were able to identify IL-33 release after trauma as an initial event in the inflammatory process of direct ARDS, initial IL-33 levels did not provide sufficient statistical reliability as a predictor of ARDS in polytrauma care. However, considering our findings, we advocate the option of a damage control approach when elevated IL33 serum levels are encountered in the acute setting of polytrauma treatment. Furthermore, initial IL-33 levels might serve as an indicator of impending death in polytraumatized patients already at admission. Being aware of the fact that our cut-off value can only be considered as an indicative parameter due to the small number of fatalities in our study group, we recommend large multi-center trials in order to provide a reliable cut-off value.

## Data Availability

The datasets used and/or analyzed during the current study are available from the corresponding author on reasonable request.

## Abbreviations

IL-33: Interleukin-33
sST2: Soluble suppression of tumorgenicity-2
PLI: Parenchymal lung injury
ARDS: Acute respiratory distress syndrome
ISS: Injury Severity Score
AIS: Abbreviated Injury Scale
